# Commentary on ‘the impact of frailty on clinical outcomes of older patients undergoing enhanced recovery after lumbar fusion surgery: a prospective cohort study’

**DOI:** 10.1097/JS9.0000000000001702

**Published:** 2024-05-29

**Authors:** Ayush Anand, Abass O. Ajayi, Mahalaqua N. Khatib, Quazi S. Zahiruddin, Sarvesh Rustagi, Prakasini Satapathy

**Affiliations:** a BP Koirala Institute of Health Sciences, Dharan, Nepal; b Ahmadu Bello University Teaching Hospital, Zaria, Nigeria; c MediSurg Research, Darbhanga; d Division of Evidence Synthesis; e South Asia Infant Feeding Research Network (SAIFRN), Division of Evidence Synthesis, Global Consortium of Public Health and Research, Datta Meghe Institute of Higher Education, Wardha; f School of Applied and Life Sciences, Uttaranchal University, Dehradun, Uttarakhand; g Center for Global Health Research, Saveetha Medical College and Hospital, Saveetha Institute of Medical and Technical Sciences, Saveetha University, Chennai, India; h Medical Laboratories Techniques Department, AL-Mustaqbal University, 51001 Hillah, Babil, Iraq

*Dear Editor*,

We would like to congratulate Wang *et al*.^1^ on publication of the manuscript ‘The Impact of Frailty on Clinical Outcomes of Older Patients Undergoing Enhanced Recovery After Lumbar Fusion Surgery: A Prospective Cohort Study’. This study provides a valuable contribution to the understanding of how frailty affects surgical outcomes in the elderly population, specifically those undergoing lumbar fusion surgery. First, the study reported that a significant portion of older patients undergoing lumbar fusion surgery are frail (50.6% classified as frail)^1^. This highlights the importance of considering frailty in preoperative assessments to better predict and manage postoperative outcomes. Second, the study robustly demonstrates that frailty, as measured by the Fried frailty scale, is associated with worse postoperative outcomes. Specifically, frail patients experienced higher rates of overall adverse events (52.4 vs. 34.7%), major adverse events (17.7 vs. 7.4%), and prolonged length of hospital stay compared to nonfrail patients^1^. Third, fried frailty, along with the Mini-Nutritional Assessment Short Form scores below 12 and a higher number of fused levels (≥3), were identified as independent predictors of a prolonged hospital stay^1^. Additionally, a higher Charlson Comorbidity Index was an independent predictor for 90-day major complications^1^. Fifth, the study reported significant correlations between frailty and impaired activities of daily living, instrumental activities of daily living, and malnutrition. This underscores the multifaceted nature of frailty, which encompasses not just physical but also functional and nutritional deficits.

Despite the robustness of the study, there are a few limitations. First, the results are based on a single-center study, which may limit the generalizability of the findings to other settings or populations. Multicenter studies are needed to confirm these results across diverse healthcare settings. Second, the study does not include a control group of frail patients who received conventional care instead of ERAS protocols. Including such a comparison could have provided a clearer picture of how much ERAS protocols contribute to improved outcomes in frail patients. Third, the follow-up period primarily focused on the immediate postoperative outcomes within 90 days. Long-term follow-up would be valuable to understand the sustained impact of frailty on recovery and quality of life over time. Fourth, the study groups nonfrail and prefrail patients together, which might mask some effects that are specific to prefrail patients.

Future studies are warranted to ensure integration of the findings of the study into surgical practice. First, future studies should extend the follow-up period to at least one year to assess long-term functional and health-related quality of life outcomes in frail versus nonfrail older adults undergoing lumbar fusion surgery. Second, there is a need for intervention studies that focus on prehabilitation strategies tailored to frail patients. These should aim to enhance nutritional status, physical function, and cognitive performance preoperatively to improve surgical outcomes. Third, conducting multicenter studies involving different healthcare settings and including a more diverse patient population can help validate and generalize the findings. Fourth, future research should include a comparison group of frail patients undergoing traditional care to delineate the specific benefits of ERAS protocols for this vulnerable population. Fifth, more detailed studies on the specific components of rehabilitation programs that are most effective for frail patients postsurgery would be beneficial. This could help tailor postoperative care to maximize recovery and independence. Fifth, exploring the predictive value of different frailty scales and comparing them could help determine the most effective tools for preoperative assessment in spinal surgery contexts.

In conclusion, this study by Wang *et al*. significantly advances our understanding of the impact of frailty on surgical outcomes in the elderly undergoing lumbar fusion surgery. By identifying frailty as a key predictor of adverse outcomes and prolonged hospital stay, the study underscores the need for comprehensive preoperative assessments and tailored interventions to mitigate these risks and improve recovery trajectories for frail older adults.

## Ethical approval

Ethical approval is not applicable for this correspondence article.

## Consent

Informed consent is not applicable for this correspondence article.

## Source of funding

Not applicable.

## Author contribution

A.A. and A.O.A.: conceptualization, project administration, supervision, validation, writing – original draft, and writing – review and editing; M.N.K., Q.S.Z., S.R., and P.S.: writing – original draft and writing – review and editing.

## Conflicts of interest disclosure

No conflict of interest to declare.

## Research registration unique identifying number (UIN)

Not applicable.

## Guarantor

Abass Oluwaseyi Ajayi.

## Data availability statement

Not applicable.

## Provenance and peer review

Not commissioned, externally peer-reviewed.
